# Particulate Air Pollution, Clock Gene Methylation, and Stroke: Effects on Stroke Severity and Disability

**DOI:** 10.3390/ijms21093090

**Published:** 2020-04-27

**Authors:** Laura Cantone, Eleonora Tobaldini, Chiara Favero, Benedetta Albetti, Roberto M. Sacco, Giuseppe Torgano, Luca Ferrari, Nicola Montano, Valentina Bollati

**Affiliations:** 1 EPIGET LAB, Department of Clinical Sciences and Community Health, Università degli Studi di Milano, 20122 Milan, Italy; laura.cantone@unimi.it (L.C.); chiara.favero@unimi.it (C.F.); benedetta.albetti@unimi.it (B.A.); luca.ferrari@unimi.it (L.F.); 2 Department of Clinical Sciences and Community Health, University of Milan, 20122 Milan, Italy; eleonora.tobaldini@unimi.it (E.T.); roberto.mr.sacco@gmail.com (R.M.S.); nicola.montano@unimi.it (N.M.); 3 Department of Internal Medicine, Fondazione IRCCS Ca’ Granda Ospedale Maggiore Policlinico, 20122 Milan, Italy; 4 Department of Anesthesia, Critical Care and Emergency, Fondazione IRCSS Ca’ Granda, Ospedale Maggiore Policlinico, 20122 Milan, Italy; giuseppe.torgano@gmail.com

**Keywords:** air pollution, stroke, clock gene methylation, NIHSS, Rankin

## Abstract

Circadian rhythm disturbances have been consistently associated with the development of several diseases, particularly cardiovascular diseases (CVDs). A central clock in the brain maintains the daily rhythm in accordance with the external environment. At the molecular level, the clock is maintained by “clock genes”, the regulation of which is mainly due to DNA methylation, a molecular mechanism of gene expression regulation, able to react to and be reprogrammed by environmental exposure such as exposure to particulate matter (PM). In 55 patients with a diagnosis of acute ischemic stroke, we showed that PM_2.5_ exposure experienced before the event influenced clock genes methylation (i.e., circadian locomotor output cycles protein kaput *CLOCK*, *period 2 PER2*, *cryprochrome 1 CRY1*, *Neuronal PAS Domain Protein 2 NPAS2*), possibly modulating the patient prognosis after the event, as *cryptochrome 1 CRY1* and *period 1 PER1* methylation levels were associated with the Rankin score. Moreover, if PM_2.5_ annual average was low, CRY1/CRY2 methylation levels were positively associated with the National Institutes of Health Stroke Scale (NIHSS) score, whereas they were negatively associated if PM_2.5_ exposure was high. Whether epigenetic changes in clock genes need to be considered as a prognostic marker of stroke or rather a causal agent in stroke development remains to be determined. Further studies are needed to determine the role of clock gene methylation in regulating the response to and recovery after a stroke event.

## 1. Introduction

All living organisms display behavioral and biochemical oscillations over a 24 h period. These circadian rhythms are evolutionarily preserved and are driven by the necessity to synchronize biological activity with the ever-changing, but predictable environment of the rotating Earth [1]. Circadian clocks cause self-sustaining, cell-autonomous fluctuations across a time period of approximately 24 h (circa diem, approximately one day) [2]. Circadian rhythms are not only stimulus-evoked responses, since they continue in the absence of external signals and they can adjust to local time [3].

An increasing amount of evidence shows that interference with circadian rhythms might play a key role in the development of several diseases [4]. In particular, disruption of the timing of biological functions that are normally synchronized [5], incorrect timing of food intake [6], and sleep loss/disturbance [7,8] have been associated with disease onset.

Recently, growing interest has been focused on the role of clock genes in the pathogenesis and progression of cardiovascular disease (CVD), the leading cause of death in a majority of countries, representing a substantial public health threat. Two main clocks, a central one and a peripheral one, regulate all biological functions, including cardiovascular function such as heart rate and blood pressure [9]. It has been suggested that a loss of synchronization between the central and peripheral clocks may underlie the onset and progression of CVDs [10,11], while their role in cerebrovascular diseases and the possible link with an autonomic nervous system derangement in acute stroke is still unknown.

The molecular basis of circadian rhythms and the complex pathway regulating them involves a positive (+) and a negative (−) interconnected feedback loop modulating “clock gene” expression. In the positive loop, the transcriptional–translational feedback loop (TTL), the transcriptional activators ARNTL (Aryl hydrocarbon receptor nuclear translocator-like protein 1) dimerize with CLOCK, or possibly with neuronal NPAS2 proteins in brain tissue [12,13], and this heterodimer binds to the promoter elements (CACGTG) present in clock and clock-controlled genes (CCGs) [12,13]. In the negative loop, the clock genes Period (*PER1*, *PER2*, and *PER3*) and Cryptochrome (*CRY1* and *CRY2*), when activated by the CLOCK–ARNTL heterodimer, constitute the negative portion of the TTL: PER1, PER2, PER3, CRY1, and CRY2 proteins form heterodimers that eventually enter the nucleus to inhibit transcription by binding to the CLOCK–ARNTL complex [12,13].

The regulation of clock genes is mainly due to DNA methylation [14], one of the mechanisms of gene expression regulation, which is able to react to and be reprogrammed by environmental stimuli, such as particulate matter (PM) [15,16].

PM exposure has been associated with all-cause mortality in several studies, and the strongest associations have been repeatedly reported for CVDs [17,18,19]. Thus, the health effects of PM on CVDs are of particular value for health risk assessment. Ischemic heart disease (IHD) and stroke constitute the major diagnoses contributing to the CVD burden, and contribute to both increased morbidity and mortality [20].

To the best of our knowledge, only one study has previously reported the effects of PM exposure on the methylation pattern of genes in the circadian pathway (clock genes), in a cohort of pregnant women and their newborns [21].

In the present study, we evaluated the effects of PM_2.5_ exposure experienced before stroke on clock genes’ methylation levels, in order to investigate their possible role in modulating patients’ prognosis after the event. We developed a cross-sectional study investigating the effects of clock gene methylation and particulate air pollution on patients residing in the Lombardy region of Italy and admitted to the Emergency Department (ED) of the Fondazione IRCCS Ca’ Granda, Ospedale Maggiore Policlinico (Milan, Italy) with a diagnosis of acute ischemic stroke. We enrolled 55 incident acute ischemic stroke cases, collected within 4.5 h from the onset of neurological symptoms.

## 2. Results

### 2.1. Study Population, DNA Methylation Levels, and Exposure Levels

The study population included 55 subjects with BMI 24.8±3.8 kg/m^2^, aged 74.6 ± 13.9 years; 56.4% were males and 43.6% females and 67.3% had a previous diagnosis of hypertension (blood pressure: systolic 162 ± 26 mmHg and diastolic 89 ± 16). Of the group, 70.9% had no previous stroke or transient ischemic attack (TIA) and 65.5% had never smoked (Table 1).

Clinical features of subjects’ acute ischemic stroke are reported in Table 2. Mean NIHSS (National Institutes of Health Stroke Scale) score on admission was 7.5 (5, 14); a total 10.9% of strokes were of atherothrombotic origin, while 38.2% were cardioembolic in nature. The right hemisphere was affected in 22 (40.0%) patients, and the left in 23 (41.8%).

Modified Rankin score was between 0–2 in 36.4%, between 3–5 in 18.2%, and 6 in 23.6% of patients.

The distributions of the averaged DNA methylation for all studied genes, expressed as the percentage of 5-methylcytosine (%5mC), are described in Supplementary Table S1.

The distribution of ambient PM_2.5_ exposure in the week, 6 months and 1 year before the acute event is reported in Supplementary Figure S1.

### 2.2. Association between PM_2.5_ Exposure and Clock Gene Methylation

We evaluated the association between PM_2.5_ exposure across different time periods (individual days from −1 to −7, 6 month average, and 1 year average) and methylation level of clock genes. Supplementary Table S2 reports all the association coefficients.

**Figure 1:**
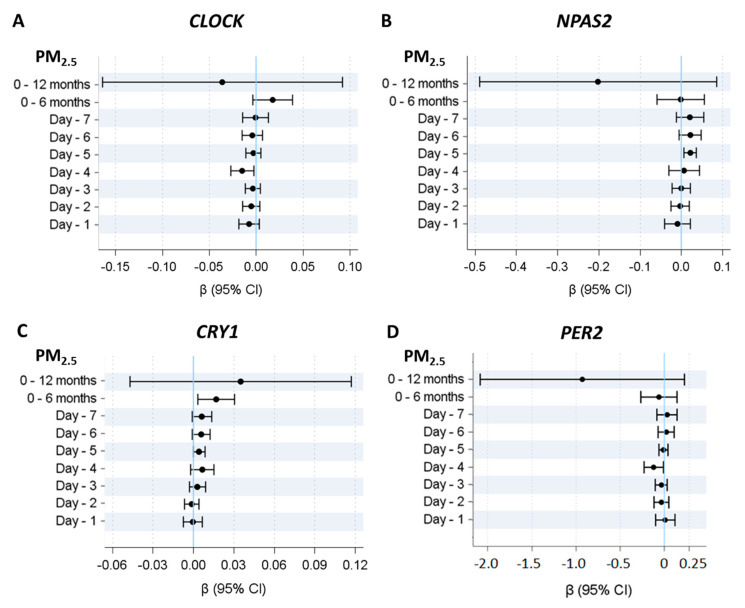
Association between PM_2.5_ exposure and clock gene methylation. Linear mixed-effect regression models were adjusted for age, sex, smoking habits, warm/cold months (months with heaters switched on/off), run, position and plate. Panel (**A**–**D**) represent beta regression coefficients and 95% CI for *CLOCK*, *NPAS2*, *CRY1*, and *PER2* methylation, respectively.

Considering the association between PM_2.5_ exposure at different time lags (individual days from −1 to −7, 6 month average, and 1 year average) and methylation level of clock genes, we observed that *CLOCK*, *PER2*, *CRY1*, and *NPAS2* were associated with PM_2.5_ levels in the 4–5 days before the event (Figure 1). In particular, *CLOCK* and *PER2* were negatively associated with PM_2.5_ exposure in the 4 days before the stroke event, (P_4days_ = 0.018 for *CLOCK* and P_4days_ = 0.032 for *PER2*), while *CRY1* and NPAS2 were positively associated with the PM_2.5_ exposure in the 5 days before the stroke event (P_5days_ = 0.046 for *CRY1* and P_5days_ = 0.009 for *NPAS2*). *CRY1* also showed long-term significant association with the 6 month average of PM_2.5_ exposure before the event.

### 2.3. Association between PM_2.5_ Exposure and NIH Stroke Scale (NIHSS) or Modified Rankin Scale Score for Neurological Disability

PM_2.5_ exposure at different time points (individual days from −1 to −7, 6 month average, and 1 year average) was not associated with NIHSS score evaluated at admission, nor was it associated with Modified Rankin Scale for Neurological Disability score.

Association coefficients are reported in Supplementary Table S3.

### 2.4. Association between Clock Gene Methylation and NIH Stroke Scale (NIHSS) Score

We further investigated the possible association between clock gene methylation and the NIHSS score evaluated at admission, and we observed that *ARNTL* methylation was negatively associated with NIHSS score (∆% = −2.2, 95% CI −4.13; −0.27, *p*-value= 0.026) (Table 3).

**Figure 2:**
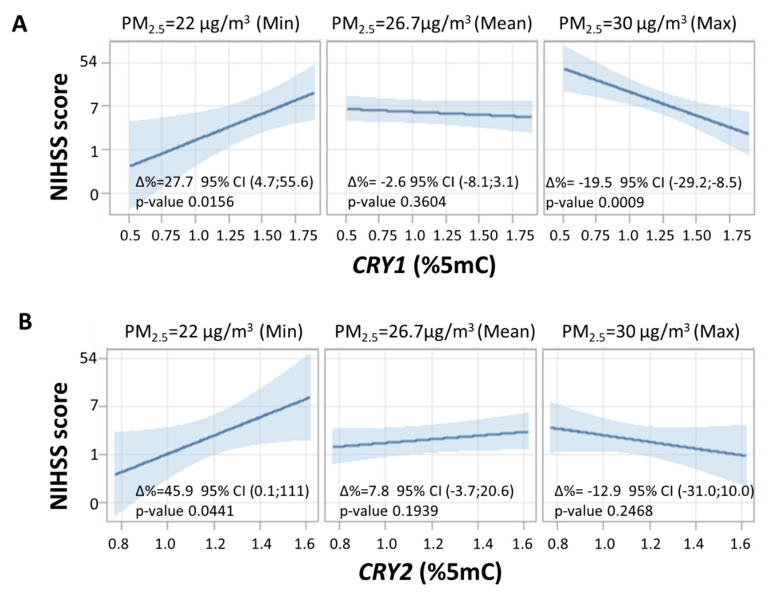
Interaction effect of PM_2.5_ annual average and clock gene methylation on NIHSS score. Strength of association between clock gene methylation and NIHSS score on natural logarithmic scale at three selected levels of annual average PM_2.5_ (min, mean, and max). Panels (**A**,**B**) represent *CRY1* and *CRY2*, respectively. For all panels, ∆% is equal to (exp(β/10)–1) × 100 and represents the percentage increase in NIHSS score for 0.15mC% increase in *CRY1/CRY2* methylation at each concentration of annual average PM_2.5_. Linear regression models were adjusted for age, sex, smoking habits, diabetes, hypertension, plate, and warm/cold months (months with heaters switched on/off).

However, when the interaction between clock gene methylation and long-term exposure to PM_2.5_ (1 year average) was considered, we observed a strong modifying effect of PM_2.5_ in modulating the association between clock gene methylation and NIHSS score. The interaction test formally performed to assess effect modification was statistically significant for *CRY1* (*p*-value = 0.031) and *CRY2* (*p*-value = 0.036). Thus, the association between *CRY1/CRY2* and the NIHSS score was modified by long-term PM_2.5_ exposure. The strength of this association in terms of percentage increase in NIHSS score observed for each 0.1% rise in *CRY1/CRY2* methylation percentage at three fixed levels of 1 year PM_2.5_ average (minimum: 22 µg/m^3^, mean: 26.7 µg/m^3^, maximum: 30 µg/m^3^) is reported in Figure 2. At low concentrations of PM_2.5_ (22 µg/m^3^), an NIHSS score increase of 27.7% for each 0.1% increment in *CRY1* methylation and an NIHSS score increase of 45.9% for each 0.1% increment in *CRY2* methylation (∆% = 27.7, 95%CI 4.7;55.6, *p*-value= 0.0156 and ∆% = 45.9, 95% CI 0.1;111, *p*-value= 0.0441, respectively) was observed. For high levels of PM_2.5_ (30 µg/m^3^), we observed a decrease of 19.5% in NIHSS score for each 0.1% increment in *CRY1* methylation (∆% = −19.5, 95% CI −29.2;−8.5, *p*-value = 0.0009), while the association between *CRY2* methylation and NIHSS score was not significant (∆% = −12.9, 95% CI −31.0;10.0, *p*-value = 0.2468). At mean concentrations of PM_2.5_ (26.7 µg/m^3^) the association was not significant for CRY1 or for CRY2 (∆% = −2.6, 95% CI −8.1;3.1, *p*-value = 0.3604 and ∆% = 7.8, 95% CI −3.7;20.6 *p*-value= 0.1939, respectively).

### 2.5. Association between Clock Gene Methylation and Modified Rankin Scale for Neurological Disability

**Figure 3:**
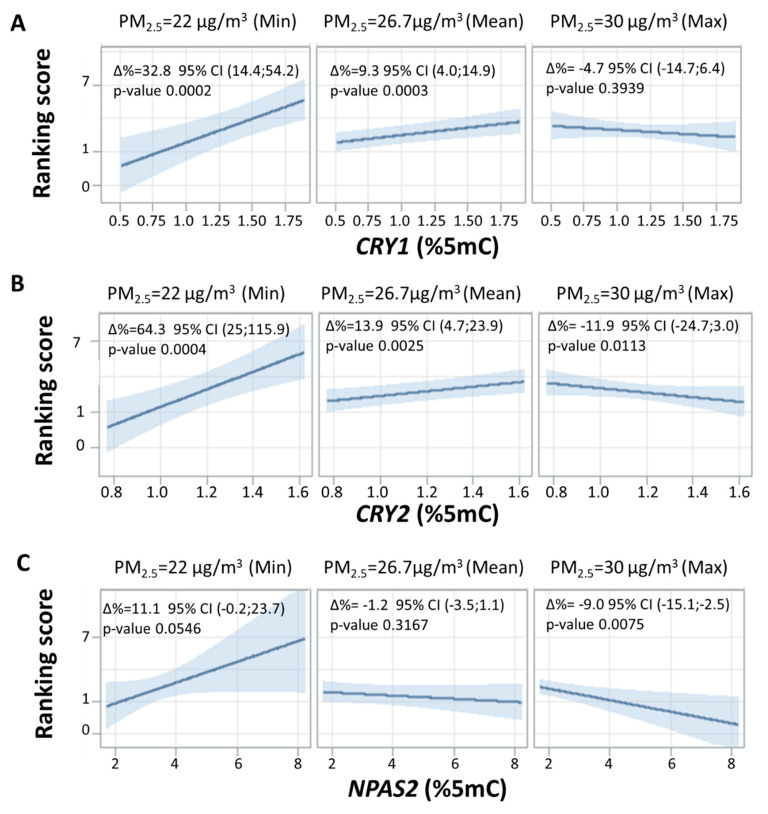
Interaction effect of PM_2.5_ annual average and clock gene methylation on Modified Rankin Scale for Neurological Disability. Strength of association between clock gene methylation and Rankin score on natural logarithmic scale at three selected levels of annual average PM_2.5_ (min, mean, and max). Panels (**A**–**C**) represent *CRY1*, *CRY2*, and *NPAS2*, respectively. For all panels, ∆% is equal to (exp(β/10)–1) × 100 and represents the percentage increase in Rankin score for 0.15mC% increment in *CRY1/CRY2/NPAS2* methylation at each concentration of annual average PM_2.5_. Linear regression models were adjusted for age, sex, smoking habits, therapy, diabetes, hypertension, plate, and warm/cold months (months with heaters switched on/off).

As reported in Table 3, *CRY1* methylation was positively associated with Rankin score 3 months after the event (∆% = 8.4, 95% CI 1.9;15.3, *p*-value= 0.010), while *PER1* was negatively associated with the Rankin score (∆% = −3.1, 95% CI –6.0;−0.14, *p*-value= 0.041). As we applied an approach similar to the one used for the NIHSS score, we observed a strong modifying effect of 1 year average PM_2.5_ in modulating the association between clock gene methylation and Rankin score (Figure 3). In particular, the formal interaction test was statistically significant for *CRY1* (*p*-value = 0.007), CRY2 (*p*-value = 0.002), and NPAS2 (*p*-value = 0.021). At low levels of PM_2.5_ (22 µg/m^3^), we observed an increase of Rankin score for each 0.1 5mC% increment in *CRY1, CRY2*, and *NPAS2* (∆% = 32.8, 95% CI 14.4;54.2, *p*-value =0.0002, ∆% = 64.3, 95% CI 25;115.9, *p*-value =0.0004 and ∆% = 11.1, 95% CI −0.2;23.7, *p*-value= 0.0546, respectively). In contrast, at high levels of PM_2.5_ (30 µg/m^3^), *CRY1, CRY2,* and *NPAS2* were associated with a decrease in the Rankin score (∆% = −4.7, 95% CI −14.7;6.4, *p*-value= 0.3939, ∆% = −11.9, 95% CI −24.7;3.0, *p*-value= 0.0113 and ∆% = −9.0, 95% CI −15.1;−2.5, *p*-value= 0.0075, respectively).

## 3. Discussion

According to a recent report from the Global Burden of Disease, the estimated global lifetime risk of stroke in 2016 for those aged 25 years or older was 24.9%, an increase from the estimated 22.8% in 1990. Notably, stroke remains the second leading cause of death worldwide, with 5.5 million (95% uncertainty interval (UI) 5.3–5.7) deaths attributed to this cause in 2016 [22]. Moreover, the prevalence of stroke is expected to increase.

Among the stroke risk factors, smoking, poor diet, and physical inactivity accounted for 66%, while environmental risks (air pollution and lead exposure) accounted for about 28%.

Short-term exposure to PM has been associate with stroke incidence [20], as well as with increased mortality in stroke patients [23].

An increasing amount of evidence shows that the disruption of circadian rhythms might contribute to the development of stroke [24]. In particular, stroke has a 24 h rhythm related to incidence and disease burden, as stroke onset increases in frequency upon awakening in humans between 6 AM and 12 PM [25]. These variations have been explained as being due to circadian variation in the autonomic nervous system [26] or to relevant cardiovascular risk factors that are regulated by peripheral circadian clocks, such as platelet aggregation [27].

In the present study, we showed that clock gene methylation was modified by short-term PM_2.5_ exposure. Moreover, DNA methylation of some of the clock genes we investigated (i.e., *CRY1*, *PER1*) was associated with Rankin score. Finally, if the annual average of PM_2.5_ was low, the association between *CRY1/CRY2* methylation and the NIHSS score was significant and positive, whereas it became negative and significant if PM_2.5_ exposure was high. A similar behavior was also observed for the Rankin score as, if annual average of PM_2.5_ was low, the association with *CRY1/CRY2/NPAS2* was significant and positive, whereas it became negative and significant if PM_2.5_ exposure was high.

The investigation of clock gene methylation in response to environmental stimuli is still in its infancy. To the best of our knowledge, the only available paper on this topic was published by our research group, in a population of mother–infant pairs from the ENVIRonmental influence ON Italy AGEing (ENVIRONAGE) birth cohort. The key finding of this study was that PM_2.5_ influences the methylation pattern of genes in the circadian pathway. In particular, we observed an increased methylation of *NPAS2*, *CRY1*, *PER2*, and *PER3* with an increase in 3rd trimester PM_2.5_ exposure [21]. Although the experimental conditions of this previous investigation were very different from those of the present paper, the increase of methylation in *CRY1* and *NPAS2* was coherent in the two studies, while stroke patients showed a decrease in *PER2* methylation, which was not present in healthy placental tissues.

The modification effect of long-term exposure to PM on clock gene methylation is particularly intriguing, as we can speculate that it might be a proxy of the inflammatory status of the patients. In vitro and in vivo animal studies suggest that C-reactive protein (CRP) levels increase in response to PM exposure, but there is no consistency in epidemiological studies. In the present study, CRP levels were within the normal range. A possible explanation could be the consumption of anti-inflammatory drugs, especially in subjects affected by chronic conditions. However, long-term exposure to PM has been consistently associated with increased level of low-grade inflammation, supporting the biological link between PM exposure and deteriorating cardiovascular health. Moreover, chronic exposure to PM has been linked to increased fibrinogen, which plays a key role in the clotting cascade. Fibrinogen has procoagulant and pro-inflammatory properties, and promotes atherothrombosis [28].

A second hypothesis is that, as clock genes are modulated by short-term PM_2.5_ exposure, long-term exposure might cause a sort of “adaptation”, which makes certain patients less responsive to short-term PM_2.5_ exposure and therefore less able to hypermethylate clock genes.

From the clinical point of view, we could speculate that this biomarker could help the general approach to patients with acute stroke. For instance, it could be helpful for a better stratification of stroke patients at higher risk in the acute setting. In addition, it could also be possible to use this marker to identify specific patterns of response to acute treatments such as thrombolysis. Finally, a possible prognostic role could help to identify the best treatment strategy for these patients.

The present study must be interpreted taking into account both its strength and its limitations. First, although the number of recruited patients was limited, the inclusion criteria of this study were very strict, allowing us to consider a very homogenous group of patients; all were recruited in the same hospital, within 4.5 h from the onset of disease, and before any clinical intervention. Moreover, we collected very detailed information about possible confounding factors and we were able to consider them in the statistical analyses. Second, in this study, we investigated the effects of PM_2.5_ as the principal exposure. However, air pollution involves exposure to a complex mixture of gaseous and particulate environmental pollutants, such as fine and ultrafine particles, nitrogen dioxide, ozone, and sulfur dioxide. Therefore, we cannot exclude effects from other pollutants as well as interactions between different pollutants.

Third, all the methylation analyses in this study were conducted on buffy coat. Although PM enters the body through the airway, which is the first body district impacted by this type of exposure, several studies have demonstrated that PM exposure affects DNA methylation in blood cells, and the majority of the study in the field have been conducted as bulk analyses by evaluating buffy coat samples [29,30,31].

The interplay between long-term exposure, *CRY* methylation, and NIHSS score raises new questions on the possible role of chronic exposure in modulating DNA methylation. This effect might thus be associated with patient prognosis following stroke. Future studies investigating gene expression and focusing on changes in DNA methylation occurring over time are required, together with those aimed at unveiling the role of clock gene methylation in regulating the response to and recovery after a stroke event.

Our findings are promising, although based on a small sample mainly due to the difficulty in recruiting this type of subject. Thus, further studies on larger populations, such as multicenter studies, are warranted in order to confirm the evidence described here.

## 4. Materials and Methods

### 4.1. Study Population

From September 2016 to March 2018, we enrolled 55 patients admitted to the Emergency Department (ED) of Fondazione IRCCS Ca’ Granda, Ospedale Maggiore Policlinico (Milan, Italy) with a diagnosis of acute ischemic stroke and presenting within 4.5 h from the onset of neurological clinical symptoms. Inclusion criteria were: age > 18, an ischemic origin of stroke, onset of symptoms in the previous 4.5 h, symptoms present at least for 30 min without any clinically relevant improvement, and absence of any significant mass effect resulting in a shift of midline. Exclusion criteria were: primary intracerebral hemorrhage, absence of stable spontaneous sinus rhythm on the ECG at presentation, clinically relevant pre-existing neurological deficit, generalized seizures at the onset of stroke symptoms, severe organ failure, cerebral or extracerebral cancer disease with reduced life expectancy, mechanical ventilation, or consent refusal.

For each patient, we collected demographics, anthropometrics, clinical, ongoing drug therapy, and standard laboratory evaluations performed in the Emergency Department.

All subjects gave their informed consent for inclusion before they participated in the study. The study was conducted in accordance with the Declaration of Helsinki, and the protocol was approved by the Ethics Committee “Comitato Etico—Milano Area 2” of the Fondazione IRCCS Ca’ Granda Ospedale Maggiore Policlinico, 20122 Milan, Italy (approval number 1443/2016).

Patients underwent diagnostic and therapeutic procedures according to International and Internal Guidelines for the treatment of acute ischemic stroke. Stroke severity was evaluated using the National Institutes of Health Stroke Scale (NIHSS), a scale based on the evaluation of 11 neurological items with an overall score which ranges from 0 to 42, with higher scores indicating more severe neurological impairment [32]. The Modified Rankin Scale (mRS) measures the degree of dependence in daily activities of patients with stroke [22]. The scale ranges from 0 (patient without symptoms), to 6 (patient death); a value of 5 means a patient with severe disability and requiring constant care. We considered mRS at 3 months as a medium-term outcome [33].

### 4.2. Exposure Assessment

PM_2.5_ concentrations were assigned to each participant’s residential address from September 2016 to March 2018. The PM_2.5_ concentrations were recorded by the Regional Environmental Protection Agency (ARPA Lombardia) through monitoring stations located throughout Lombardy and available online as daily means. To assign a PM_2.5_ exposure level to each subject, the addresses of the monitoring stations and the study subjects were geocoded and the PM_2.5_ measured by the nearest monitor to residential address was assigned. We chose to investigate short-term PM_2.5_ exposure (we used the daily means of PM_2.5_ concentrations measured 1 day before the date of recruitment and back to 7 days before enrollment) as well as long-term PM_2.5_ exposure (6 month and 1 year averages). In cases of incomplete series, missing values were attributed using an algorithm that integrated the annual average of the incomplete series and the PM_2.5_ concentrations of the nearest and more correlated monitors [34].

### 4.3. Sample Collection, DNA Extraction, and Bisulfite Treatment

Seven millilitres of whole blood were collected into EDTA tubes from each participant by venous phlebotomy. Blood was centrifuged at 1200 *g* for 15 min. The buffy coat fraction was transferred to a cryovial and immediately frozen at −80 °C until use. DNA was extracted using a Wizard Genomic DNA purification kit (Promega, Madison, WI, USA) according to the manufacturer’s instructions. Next, 500 ng of genomic DNA was treated with the EZ DNA Methylation-Gold™ Kit (Zymo Research, Orange, CA, USA) in accordance with the manufacturer’s protocol. Bisulfite-treated DNA was eluted in 30 μL of M-Elution Buffer and kept at −80 °C until use.

### 4.4. DNA Methylation

Analysis of DNA methylation was performed via previously published methods [21,35,36] with minor modifications. Briefly, a 50 μL PCR reaction was carried out with 25 μL of Hot Start GoTaq Green Master mix (Promega), 1 pmol of forward primer, 1 pmol of biotinylated reverse primer, and 25 ng of bisulfite-treated genomic DNA. Biotin-labeled primers were used to purify the final PCR product with sepharose beads. The PCR product was bound to a Streptavidin Sepharose HP (Amersham Biosciences, Uppsala, Sweden). Sepharose beads containing the immobilized PCR product were purified, washed, denatured with 0.2 M NaOH, and washed again with the Pyrosequencing Vacuum Prep Tool (Pyrosequencing, Inc., Westborough, MA, USA) according to the manufacturer’s instructions. Pyrosequencing primer (0.3 μM) was annealed to the purified single-stranded PCR product, and pyrosequencing was performed with the PyroMark MD System (Pyrosequencing, Inc. Westborough, MA, USA). CpG sites were interrogated within the promoter regions of the following genes: *ARNTL*, *CLOCK*, *NPAS2*, *CRY1*, *CRY2*, *PER1*, *PER2*, *PER3*.

PCR cycling conditions and primer sequences are reported in Supplementary Table S4. The methylation level at CpG positions within each gene’s promoter region was expressed as the percentage of cytosines that were methylated, determined as the number of methylated cytosines divided by the sum of methylated and unmethylated cytosines, multiplied by 100% (% 5-methyl-cytosine). Every sample was tested twice for each marker to confirm reproducibility and to increase the precision of the findings.

### 4.5. Statistical Analysis

The data were summarized with standard descriptive statistics. Categorical variables were presented as absolute numbers and frequencies. Continuous variables were expressed as the mean ± standard deviation (SD) or as the median (first quartile–third quartile), as appropriate.

To estimate the effect of PM_2.5_ exposure on clock gene methylation, we fitted linear mixed-effect models adjusted for age, sex, smoking habits, and warm/cold months. DNA methylation measurements for each subject were run in duplicate. The pyrosequencing-based DNA methylation analysis tested a variable number of CpG positions according to CpG density in the promoter assay. Linear mixed-effect models were used to account for each CpG dinucleotide position (as a random effect) measured in the two runs and the potential confounding effect of the plate. An unstructured covariance structure was used to model within-subject errors. The Kenward–Roger approximation was used to estimate the degrees of freedom in the denominator.

We further evaluated the associations of different time of PM_2.5_ exposure (short- and long-term exposure) with NIHSS and Modified Rankin scores. We performed linear regression models adjusted for age, sex, smoking habits, diabetes, hypertension, warm/cold months, and therapy (for Modified Rankin score). Dependent variables were log-transformed (base e) to achieve normality of models’ residuals. Effects were thus expressed as ∆% with a 95% confidence interval (CI), which corresponded to (exp(β)–1) × 100 and represented the percentage increase in NIHSS or Modified Rankin score for 1µg/m^3^ increase in PM_2.5_. In considering the effect of clock gene methylation on NIHSS score and Modified Rankin score, we examined the potential effect modification of long-term PM exposure (i.e., PM_2.5_ annual average). Effects were thus expressed as ∆% with a 95% confidence interval (CI), which corresponded to (exp(β/10)–1) × 100 and represented the percentage increase in NIHSS or Modified Rankin score for a 0.1% increase in clock gene methylation.

Normality and linearity assumption were verified by graphical inspection. A *p*-value of 0.05 was considered statistically significant. All analyses were performed in SAS 9.4 (SAS Institute Inc., Cary, NC, USA).

## Figures and Tables

**Table 1:** Demographic and clinical characteristics of the studied population (*n* = 55).

Characteristics	Value
Age (years)	74.6 ± 13.9
Sex:	
Male	31 (56.4%)
Female	24 (43.6%)
BMI (kg/m^2^)	24.8 ± 3.8
Blood pressure (mmHg):	
Systolic	162 ± 26
Diastolic	89 ± 16
Glucose (mg/dL)	120.2 ± 30.9
Hypertension:	
Yes	37 (67.3%)
Diabetes:	
Yes	8 (14.5%)
Atrial fibrillation:	
Yes	17 (30.9%)
Heart failure:	
Yes	6 (10.9%)
Previous stroke or TIA:	
Yes	16 (29.1%)
Smoking status:	
Never smoked	36 (65.5%)
Current smoker	12 (21.8%)
*n*.a.	7 (12.7%)
Hb (g/dL)	13.8 ± 1.7
Ht (%)	40.1 ± 4.3
Plt × 10^3^ (N/μL)	225 (193, 304)
AST (U/L)	23.1 ± 8.8
ALT (U/L)	19.2 ± 9.1
PT	1.12 ± 0.37
aPTT	0.95 ± 0.12
Albumin (g/dL)	3.9 ± 0.4
Creatinine (mg/dL)	1.03 ± 0.34
C-reactive protein (mg/dL)	0.34 (0.15, 0.66)
Total cholesterol (mg/dL)	183 ± 34.9

Plt and C-reactive protein are expressed as median (Q1, Q3) because values were not normally distributed. Other variables are expressed as mean ± standard deviation. ALT: alanine aminotransferase; AST: aspartate aminotransferase; BMI: body mass index; PT: prothrombin time; aPTT: activated partial thromboplastin time; TIA: transient ischemic attack.

**Table 2:** Clinical features of acute ischemic stroke (*n* = 55).

Characteristics	Value
NIHSS score on admission:	7.5 (5,14)
<14	36 (65.5%)
≥14	14 (25.4%)
n.a.	5 (9.1%)
Therapy:	
Yes	31 (56.4%)
n.a.	8 (14.5%)
TOAST:	
Atherombotic	6 (10.9%)
Cardioembolic	21 (38.2%)
Lacunar	2 (3.7%)
Indeterminate	16 (29.1%)
n.a.	10 (18.1%)
Hemisphere of stroke:	
Right	22 (40.0%)
Left	23 (41.8%)
Bilateral	3 (5.5%)
n.a.	7 (12.7%)
Circle segments:	
Anterior	42 (76.4%)
Posterior	6 (10.9%)
n.a.	7 (12.7%)
Modified Rankin score at 3 months:	2.95 ±2.5
0–2	20 (36.4%)
3–5	10 (18.2%)
6	13 (23.6%)
n.a.	12 (21.8%)

NIHSS on admission is expressed as median (Q1, Q3) because values were not normally distributed. Modified Rankin scale at three months is reported as mean ± standard deviation. NIHSS: National Institutes of Health Stroke Scale; TOAST: Trial of Org 10172 in Acute Stroke Treatment (classification of stroke subtypes based on etiology as a main criterion).

**Table 3:** Association between clock gene methylation and NIH stroke scale (NIHSS) score and Modified Rankin Scale for Neurological Disability score.

Scale	Clock Gene Methylation	∆%	95% CI		*p*-Value
**NIHSS score**	***ARNTL***	**−2.22**	**−4.13**	**−0.27**	**0.0256**
	*CLOCK*	−0.13	−2.21	1.99	0.9038
	*NPAS2*	−0.88	−2.72	0.98	0.3510
	*CRY1*	−2.98	−8.65	3.05	0.3251
	*CRY2*	1.93	−9.38	14.66	0.7498
	*PER1*	0.87	−2.00	3.83	0.5550
	*PER2*	−0.03	−0.32	0.27	0.8622
	*PER3*	−0.25	−0.59	0.09	0.1543
**Modified Rankin score**	*ARNTL*	0.35	−4.37	5.31	0.8857
	*CLOCK*	1.77	−1.91	5.59	0.3493
	*NPAS2*	−1.92	−4.50	0.73	0.1537
	***CRY1***	**8.39**	**1.92**	**15.28**	**0.0103**
	*CRY2*	7.51	−2.65	18.73	0.1527
	***PER1***	**−3.12**	**−6.01**	**−0.14**	**0.0406**
	*PER2*	−0.24	−0.50	0.02	0.0756
	*PER3*	−0.25	−0.56	0.07	0.1253

Linear regression models were adjusted for age, sex, smoking habits, diabetes, hypertension, PM_2.5_ annual average, warm/cold months, plate, and therapy (only for Rankin score). Scores were log (base e)-transformed to achieve a normal distribution. ∆% is equal to (exp(β/10)–1) × 100 and represents the percentage increase in NIHSS or Modified Rankin score for 0.15mC% increase in clock gene methylation. Clock genes with a *p*-Value ≤ 0.05 are reported in bold.

## Supplementary Materials

Supplementary materials can be found at https://www.mdpi.com/1422-0067/21/9/3090/s1. Table S1. Mean levels of gene methylation (%5mC) of the studied population (*n* = 55). Table S2. Association between PM_2.5_ exposure and clock gene methylation. Table S3. Association between PM_2.5_ exposure and NIH-STROKE SCALE (NIHSS) and Modified Rankin Scale for Neurologic Disability. Figure S1. Box plot showing the distribution of PM_2.5_ concentrations for the exposure intervals defined.
