# Supplementary material for: Particulate Air Pollution, Clock Gene Methylation, and Stroke: Effects on Stroke Severity and Disability
Source: Int J Mol Sci. 2020 Apr 27;21(9):3090. doi: 10.3390/ijms21093090 (PMC7247663; doi:10.3390/ijms21093090)

Supplementary table 1. Mean levels of gene methylation (%5mC) of the studied population (N=55).

Methylation %5mC	Value
ARNTL	1.24 ± 0.81
CLOCK	1.5 ± 0.83
NPAS2	3.34 ± 1.24
CRY1	1.24 ± 0.4
CRY2	1.2 ± 0.2
PER1	1.44 ± 0.65
PER2	74.32 ± 6.71
PER3	82.71 ± 7.21

Supplementary table 2. Association between $PM_{2.5}$ exposure and clock genes methylation.

clock gene methylation	$PM_{2.5}$	β	SE	95% CI		P-value
ARNTL	Day -1	-0.0021	0.0074	-0.0171	0.0129	0.7794
	Day -2	-0.0055	0.0057	-0.0170	0.0060	0.3356
	Day -3	-0.0025	0.0050	-0.0126	0.0077	0.6233
	Day -4	-0.0134	0.0085	-0.0306	0.0039	0.1236
	Day -5	-0.0039	0.0050	-0.0141	0.0063	0.4395
	Day -6	-0.0019	0.0069	-0.0159	0.0121	0.7827
	Day -7	-0.0002	0.0086	-0.0179	0.0174	0.9778
	0 - 6 months	0.0082	0.0139	-0.0199	0.0363	0.5588
	0 - 12 months	-0.1139	0.0787	-0.2735	0.0457	0.1565
CLOCK	Day -1	-0.0075	0.0054	-0.0184	0.0033	0.1678
	Day -2	-0.0053	0.0044	-0.0142	0.0037	0.2386
	Day -3	-0.0038	0.0039	-0.0118	0.0041	0.3354
	**Day -4**	**-0.0150**	**0.0060**	**-0.0272**	**-0.0027**	**0.0180**
	Day -5	-0.0032	0.0039	-0.0113	0.0048	0.4209
	Day -6	-0.0042	0.0052	-0.0148	0.0064	0.4299
	Day -7	-0.0009	0.0067	-0.0146	0.0128	0.8909
	0 - 6 months	0.0176	0.0105	-0.0037	0.0389	0.1029
	0 - 12 months	-0.0359	0.0632	-0.1639	0.0920	0.5727
NPAS2	Day -1	-0.0096	0.0147	-0.0404	0.0212	0.5205
	Day -2	-0.0029	0.0104	-0.0248	0.0189	0.7823
	Day -3	-0.0006	0.0106	-0.0227	0.0214	0.9546
	Day -4	0.0066	0.0175	-0.0303	0.0434	0.7114
	**Day -5**	**0.0213**	**0.0073**	**0.0060**	**0.0367**	**0.0093**
	Day -6	0.0211	0.0127	-0.0055	0.0477	0.1128
	Day -7	0.0209	0.0157	-0.0121	0.0538	0.1996
	0 - 6 months	-0.0015	0.0275	-0.0589	0.0558	0.9558
	0 - 12 months	-0.2018	0.1377	-0.4891	0.0855	0.1583
CRY1	Day -1	-0.0006	0.0033	-0.0076	0.0064	0.8557
	Day -2	-0.0013	0.0026	-0.0067	0.0041	0.6264
	Day -3	0.0029	0.0029	-0.0031	0.0088	0.3250
	Day -4	0.0066	0.0041	-0.0020	0.0152	0.1260
	**Day -5**	**0.0043**	**0.0020**	**0.0001**	**0.0084**	**0.0461**
	Day -6	0.0056	0.0031	-0.0009	0.0122	0.0887
	Day -7	0.0063	0.0034	-0.0008	0.0134	0.0804
	**0 - 6 months**	**0.0169**	**0.0066**	**0.0031**	**0.0306**	**0.0189**
	0 - 12 months	0.0350	0.0393	-0.0470	0.1170	0.3834
CRY2	Day -1	0.0026	0.0021	-0.0017	0.0069	0.2205
	Day -2	-0.0012	0.0015	-0.0043	0.0019	0.4282
	Day -3	-0.0014	0.0015	-0.0045	0.0017	0.3665
	Day -4	-0.0034	0.0024	-0.0085	0.0017	0.1812
	Day -5	-0.0008	0.0011	-0.0031	0.0015	0.4770
	Day -6	0.0007	0.0020	-0.0034	0.0048	0.7234
	Day -7	0.0000	0.0024	-0.0050	0.0050	0.9849

	0 - 6 months	-0.0010	0.0040	-0.0093	0.0074	0.8117
	0 - 12 months	-0.0179	0.0207	-0.0610	0.0253	0.3975
**PER1**	Day -1	0.0011	0.0045	-0.0082	0.0103	0.8179
	Day -2	-0.0040	0.0038	-0.0117	0.0038	0.3046
	Day -3	-0.0015	0.0035	-0.0086	0.0056	0.6705
	Day -4	0.0034	0.0062	-0.0092	0.0160	0.5847
	Day -5	-0.0020	0.0028	-0.0078	0.0038	0.4867
	Day -6	-0.0013	0.0042	-0.0100	0.0073	0.7567
	Day -7	-0.0036	0.0053	-0.0145	0.0074	0.5119
	0 - 6 months	0.0064	0.0093	-0.0125	0.0253	0.4986
	0 - 12 months	-0.0302	0.0541	-0.1399	0.0796	0.5807
**PER2**	Day -1	0.0112	0.0532	-0.0969	0.1192	0.8350
	Day -2	-0.0333	0.0412	-0.1170	0.0504	0.4248
	Day -3	-0.0344	0.0333	-0.1019	0.0332	0.3089
	**Day -4**	**-0.1208**	**0.0539**	**-0.2306**	**-0.0111**	**0.0319**
	Day -5	-0.0100	0.0257	-0.0623	0.0424	0.7008
	Day -6	0.0212	0.0441	-0.0686	0.1111	0.6337
	Day -7	0.0307	0.0563	-0.0843	0.1457	0.5896
	0 - 6 months	-0.0606	0.1012	-0.2657	0.1446	0.5533
	0 - 12 months	-0.9299	0.5711	-2.0871	0.2274	0.1120
**PER3**	Day -1	0.0477	0.0807	-0.1234	0.2188	0.5628
	Day -2	0.0284	0.0566	-0.0911	0.1478	0.6228
	Day -3	0.0270	0.0573	-0.0935	0.1474	0.6439
	Day -4	0.1193	0.0917	-0.0751	0.3137	0.2118
	Day -5	-0.0036	0.0501	-0.1099	0.1027	0.9437
	Day -6	-0.0314	0.0766	-0.1931	0.1303	0.6870
	Day -7	0.0113	0.0890	-0.1773	0.2000	0.9003
	0 - 6 months	0.1376	0.1525	-0.1827	0.4579	0.3787
	0 - 12 months	0.3757	0.7720	-1.2461	1.9975	0.6324

Linear mixed models were adjusted for age, gender, smoking habits, warm/cold months (months with heaters switched on/off) run, position and plate.

Supplementary table 3. Association between $PM_{2.5}$ exposure and NIH-STROKE SCALE (NIHSS) and Modified Rankin Scale for Neurologic Disability.

	$PM_{2.5}$	Δ%	95% CI		P-value
**NIHSS score**	Day -1	0,61	-0,48	1,70	0,2627
	Day -2	0,18	-0,63	1,01	0,6483
	Day -3	-0,08	-0,81	0,65	0,8227
	Day -4	-0,61	-1,86	0,65	0,3306
	Day -5	-0,19	-0,88	0,49	0,5682
	Day -6	0,04	-0,89	0,97	0,9363
	Day -7	-0,31	-1,53	0,94	0,6166
	0 - 6 months	-1,12	-3,02	0,81	0,2428
	0 - 12 months	0,24	-11,18	13,12	0,9684
**Modified Rankin score**	Day -1	0,38	-1,01	1,80	0,5684
	Day -2	-0,10	-1,10	0,91	0,8323
	Day -3	0,75	-1,07	2,60	0,4011
	Day -4	0,41	-1,32	2,18	0,6229
	Day -5	0,60	-1,37	2,62	0,5288
	Day -6	-0,60	-2,61	1,46	0,5460
	Day -7	-0,30	-1,92	1,36	0,7077
	0 - 6 months	-1,16	-3,64	1,38	0,3462
	0 - 12 months	1,87	-14,88	21,93	0,8305

Linear regression models were adjusted for age, gender, smoking habits, diabetes, hypertension and warm/cold months (months with heaters switched on/off) and therapy (only for Ranking score). Scores were log (base e) transformed to achieve a normal distribution. Δ% is equal to (exp(β)–1)*100 and represents the percentage increase in NIHSS or Ranking score for 1 $\mu g/m^3$ increase in $PM_{2.5}$.

Supplementary Table 4. Pyrosequencing assay information. For all genes assembly hg19 was used in UCSC genome browser, with the exception of CRY1 where assembly hg18 was used.

Gene	Chromosome and region	CpG loci	Primers: Forward (F), Reverse (R) and Sequencing (S)	Sequencing length
**ARNTL**	chr11:13297365-13299374	2	F: TAGGGGATTTAGAGAAGAGGGATAT<br>R: biotin- ACTACTTTCCTACCACCAATCATTTAA<br>S: TTATTTTATTTTATTTTAGT	38 bp
**CLOCK**	chr4:56413309-56413697	3	F: TTTTTAGGAGATGGGAGAAGATGT<br>R: biotin- TAAAAAATCCAAAAACCAAAAAAAA<br>S: TTTTTTGTTAATATT	28 bp
**NPAS2**	chr2:101435534-101435968	3	F: GGGAATTTTGGTAAAATTTTTTTT<br>R: biotin- CTCCTCTCCTTTTACACCAATACAA<br>S: GGGAATTTTGGTAAAATTTTTTTT	28 bp
**CRY1**	chr12:106011400-106012370	3	F: TTTGTGAGGGAAGGTTYAGTTT<br>R: biotin- AACAATTTCCAAACCCTCC<br>S: TTTTTAAGGGTTATGAG	27 bp
**CRY2**	chr11:45868457-45869129	4	F: TGTTTTTTGAGATTTGGTTTATTTT<br>R: biotin- TAGTTAATGGTAGAGGGGTTTTGG<br>S: TGTTTTTTGAGATTTGGTTTATTTT	33 bp
**PER1**	chr17:8055042-8055979	3	F: TAGGGTTAGGGATTGGAGAATAGA<br>R: biotin- ACCCAAACAAAAAACACACTATC<br>S: GGGTTAGGAGTGTAGATTTT	27 bp
**PER2**	chr2:239196677-239199714	3	F: TGAGAAAGGTAGTATTTTTAAGG<br>R: biotin- AAAACTCCACATACCCCACAC<br>S: AGGAGGTTGTTTTGGGAGAT	34 bp
**PER3**	chr1:7844128-7845255	3	F: TGTTTGTTATTGATTGTAAAGTGAG<br>R: biotin- GTAGGGAAAGGGGGATTTAAATT<br>S: TGTTTGTTATTGATTGTAAAGTGAG	25 bp

Supplementary Figure 1. Box plot showing the distribution of $PM_{2.5}$ concentrations for the exposure intervals defined

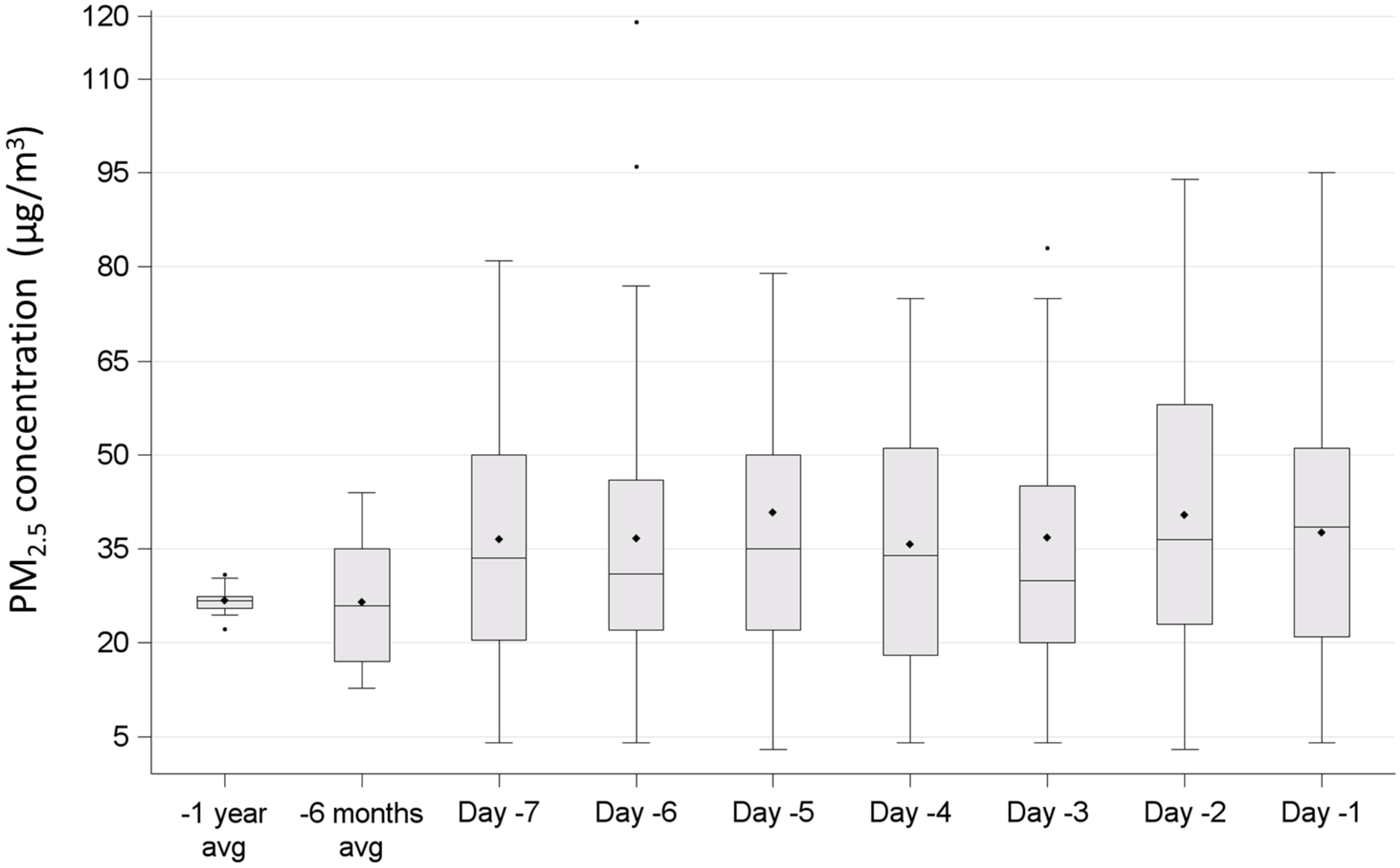

Supplement: Supplementary file 1 [file ijms-21-03090-s001.pdf]
